# Challenges in Estimating Vaccine Coverage in Refugee and Displaced Populations: Results From Household Surveys in Jordan and Lebanon

**DOI:** 10.3390/vaccines5030022

**Published:** 2017-08-12

**Authors:** Timothy Roberton, William Weiss, The Jordan Health Access Study Team, The Lebanon Health Access Study Team, Shannon Doocy

**Affiliations:** 1 Johns Hopkins Bloomberg School of Public Health, 615 N Wolfe St, Baltimore, MD 21205, USA; timroberton@jhu.edu (T.R.); wweiss1@jhu.edu (W.W.); gburnha1@jhu.edu (The Jordan Health Access Study Team); elyles@jhu.edu (The Lebanon Health Access Study Team); 2 Jordan University of Science and Technology School of Nursing, Irbid, Jordan; 3 Medecins du Monde, Beirut, Lebanon; 4 Faculty of Health Sciences, American University of Beirut, Beirut, Lebanon; 5 International Medical Corps, Beirut, Lebanon; 6 United Nations High Commissioner for Refugees, Beirut, Lebanon

**Keywords:** Syria, Jordan, Lebanon, refugee, displaced populations, humanitarian assistance, vaccination, vaccination coverage

## Abstract

Ensuring the sustained immunization of displaced persons is a key objective in humanitarian emergencies. Typically, humanitarian actors measure coverage of single vaccines following an immunization campaign; few measure routine coverage of all vaccines. We undertook household surveys of Syrian refugees in Jordan and Lebanon, outside of camps, using a mix of random and respondent-driven sampling, to measure coverage of all vaccinations included in the host country’s vaccine schedule. We analyzed the results with a critical eye to data limitations and implications for similar studies. Among households with a child aged 12–23 months, 55.1% of respondents in Jordan and 46.6% in Lebanon were able to produce the child’s EPI card. Only 24.5% of Syrian refugee children in Jordan and 12.5% in Lebanon were fully immunized through routine vaccination services (having received from non-campaign sources: measles, polio 1–3, and DPT 1–3 in Jordan and Lebanon, and BCG in Jordan). Respondents in Jordan (33.5%) and Lebanon (40.1%) reported difficulties obtaining child vaccinations. Our estimated immunization rates were lower than expected and raise serious concerns about gaps in vaccine coverage among Syrian refugees. Although our estimates likely under-represent true coverage, given the additional benefit of campaigns (not captured in our surveys), there is a clear need to increase awareness, accessibility, and uptake of immunization services. Current methods to measure vaccine coverage in refugee and displaced populations have limitations. To better understand health needs in such groups, we need research on: validity of recall methods, links between campaigns and routine immunization programs, and improved sampling of hard-to-reach populations.

## 1. Introduction

The severity and frequency of humanitarian emergencies are increasing, as are populations affected by these events: there are currently more persons displaced by conflict than at any other time in history [1]. The adverse effects of humanitarian emergencies are widespread and include increased mortality, notably due to communicable diseases [2,3]. Communicable disease prevention in humanitarian settings typically involves the provision of clean drinking water, improved sanitation, and basic primary care [4]. Equally important is the management of vaccine-preventable disease through rapid immunization campaigns. The disruption of a country’s routine immunization services, combined with an exacerbation of factors associated with disease transmission (mass population movement, overcrowding, malnutrition), can increase the likelihood of fatal disease outbreaks [5]. Recently the re-emergence of polio was seen in Syria, along with measles outbreaks in neighboring areas [6]. To prevent such outbreaks, rapid immunization campaigns are recommended in the immediate aftermath of an acute humanitarian emergency [7]. Measles and polio vaccinations are the most consistently recommended campaigns [4,7,8]. Despite these recommendations, some suggest that immunization campaigns are underutilized in humanitarian settings, warning that this not only costs lives but sets back global disease eradication and elimination efforts [6,9].

Even if campaigns fill gaps in provision of measles and polio vaccinations, refugee and displaced children living outside of camps in a host country may not receive other routine vaccinations that are also important to ensure health, such as vaccinations for tuberculosis, diphtheria, tetanus, whooping cough, hepatitis B, haemophilus influenzae type B, or pneumococcal pneumonia. Essential health services may be provided for children in camps, but many refugee and displaced families live outside of camps and do not have access to these in-camp services. Families living outside of camps may also struggle to access services from the host country health system or other providers. As part of a comprehensive humanitarian response, we should ensure that all children have their full schedule of vaccinations.

Vaccination coverage—the proportion of children needing a vaccine who have, in reality, received that vaccine—is the global standard for understanding met need for vaccines and the success of vaccination programs [10]. Survey methodologies for estimating vaccination coverage have been established [11,12,13]. Estimates from household surveys are perceived as more reliable than those generated from other sources such as administrative data [14,15,16,17]; however, the use of household surveys to estimate vaccination coverage has limitations. Information bias is a concern, as vaccination cards may be difficult to read or absent, and mother’s recall of vaccinations can be unreliable [10,18]. In humanitarian settings, household surveys are further vulnerable to sampling bias due to lack of accurate data for developing sampling frames.

More often than not, vaccination coverage in humanitarian settings is estimated through post-campaign surveys. These have high reliability because caregivers are better able to recall vaccines that were recently administered. However, post-campaign surveys only measure coverage of the vaccine distributed in the campaign. Fewer surveys measure coverage of all the vaccines that children are expected to receive, though understanding full vaccination coverage provides a better snapshot of public health risks and the success or failure of the health system.

In 2014 and 2015 we conducted surveys of Syrian refugees in Jordan and Lebanon living outside of camps, including refugees that were and were not registered with the UN Refugee Agency (UNHCR). The aim of both studies was to assess health access and utilization, and one measure included in the surveys was vaccination coverage. In this paper we report vaccination rates of Syrian refugee children 12–23 months of age living outside of camps, assess the reliability of the results, and highlight ways in which the methodology we employed could have introduced bias. We suggest that these challenges are not specific to our study but are inherent in measuring vaccination coverage in refugee and displaced populations.

## 2. Methods

Two cross-sectional surveys of Syrian refugees were conducted to characterize health-seeking behaviors and better understand issues related to accessing health services; first in Jordan in June 2014, and then in Lebanon in March and April 2015. For both surveys, a two-stage cluster survey design with probability proportional to size sampling was used to attain a nationally representative sample of Syrian refugees living outside of camps [19]. Sample size was determined for key study objectives based on the most conservative prevalence rate estimate of 50%; calculations assumed 80% power and a design effect of 2.0 to account for the cluster sample design. The sample size for each country was further increased to 1400 refugee households to account for up to a 10% non-response rate, and to increase precision of point estimates and additional power for the detection of statistically significant differences of >10% for comparing key indicators between registered and unregistered Syrian refugees and Syrian refugees in different sub-national regions.

**Figure 1:**
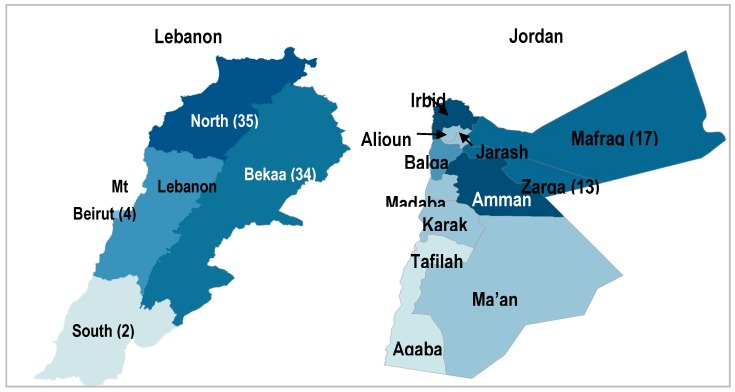
Cluster assignment by governorate.

Given the concentration of Syrian refugees and the low cost of visiting many locations due to the countries’ small sizes, a 125 cluster × 12 household design was chosen in Jordan and a 100 cluster × 14 household design was used in Lebanon. Probability proportional to size sampling was used to assign clusters to cadastrals using registration data from UNHCR, assuming that non-registered refugees had similar residence patterns. In Lebanon, the research team was unable to attain permission to conduct the survey in certain security-sensitive areas as planned, which necessitated a re-draw of the 28 clusters that were originally assigned to 22 inaccessible cadastrals. Clusters were re-assigned using probability proportional to size sampling methods based on a revised population residing in accessible areas [19]. Cluster assignment by governorate in both countries is shown in Figure 1.

Different sampling strategies were used to identify respondents in Jordan and Lebanon. In each cluster of the Jordan survey, UNHCR randomly selected five registered refugee households that were listed as living in that cluster’s assigned sub-district. Households were then called by the study team; the first household that was currently residing in the specified sub-district and agreed to meet with the study team was used as the index household for the cluster. The study team met this household, conducted an abbreviated interview (the results of which were not included in the survey data set to minimize bias towards registered refugees), and enquired about Syrian households living in the vicinity. The household(s) to which the index household referred the interview teams were then interviewed using the complete questionnaire. This methodology was adopted to ensure that both registered and unregistered refugees living outside of camps were included in the sample. (The authors used a similar technique in previous studies to sample Iraqi refugees living among host populations [20].) In both the Jordan and Lebanon surveys, household heads and primary caretakers of children were prioritized as respondents and answered questions on behalf of the households and its members.

In Lebanon, once clusters were assigned to the cadastrals, ARC GIS software was used to randomly allocate cluster start points. Coordinates in developed areas were used and the nearest intersection to the randomly identified start point, usually within a half-kilometer, was used as the cluster start point. Teams were provided with coordinates to locate each cluster’s start point and maps with satellite imagery of the area. After navigating to the start point, interviewer pairs were sent in different directions to locate households. As the interviewers walked in the assigned direction, they approached the nearest business likely to be used by Syrian households such as a food shop, pharmacy, or cell phone shop, and asked to be referred to nearby Syrian households. When interviewers reached a Syrian household that consented to participate, the first interview in the cluster was conducted; upon completion of the interview, respondents were asked to provide a referral to the nearest Syrian household. This referral process was used until 14 Syrian refugee interviews were completed.

Only Syrian households arriving in the respective host country in 2011 or later were eligible to participate, as the aim of the survey was to capture the experiences of those displaced by the recent conflict. However, only one of the households approached for interview arrived in Lebanon before 2011 and none of the households approached in Jordan arrived prior to 2011.

The questionnaire was first developed for use in Jordan and adapted to the Lebanese context by consensus between partner organizations, with the aim of providing a comprehensive assessment of the health of Syrian refugees that could inform humanitarian assistance planning at local, national, and regional levels. The final questionnaire focused on health service utilization, access and barriers to care, children’s health, and chronic medical conditions. The Arabic translation of the Jordan questionnaire was checked and adapted for the Lebanese context, and formal pilot tests were performed in both countries.

Data collection teams received two days of classroom training that focused on the questionnaire, e-data collection using tablets, interview techniques, basic principles of human subjects’ protections and sampling methods. Following classroom training, additional practical field training was held. Verbal consent was obtained from all respondents. For each household with a child aged 12–23 months, respondents were asked to present the child’s EPI vaccination card. If more than two children aged 12–23 months were in the household, one child was selected at random by the interviewer, either by flipping a coin (for two children) or by spinning a pen (for three or more children). EPI cards from both Syria and the host country were accepted. For children whose EPI card was available, vaccinations were recorded by directly observing the EPI card. For children without cards or whose cards were not available, respondents were asked to recall whether the child had received each vaccination. Data were collected on tablets using the Magpi mobile data platform by DataDyne LLC (Washington, DC, USA).

The Jordan study was reviewed by ethics committees at the World Health Organization, Jordan University of Science and Technology, and Johns Hopkins School of Public Health, and was approved by the Jordanian Ministry of Health. The Lebanon study was approved by the Institutional Review Board at the American University of Beirut.

### Data Analysis

Data were analyzed using Stata 13 (College Station, TX, USA) and Tableau Desktop (Seattle, WA, USA) software packages. We generated standard descriptive statistics for comparison of means and proportions, on topics including: demographics of sample respondents; EPI cards; routine vaccination rates; difficulties obtaining vaccinations; and location of vaccination (Lebanon only). The primary unit of analysis was the child, not the household. The Stata ‘svy’ command was used to account for the cluster survey design so that standard errors of the point estimates were adjusted for survey design effects.

In addition to the above statistics, we conducted a supplementary analysis to estimate vaccine coverage from routine sources and campaigns. It was not our goal in this paper to estimate vaccination rates from campaigns, since only minimal data were collected about campaigns in the surveys, but we felt it was useful to estimate the additional coverage provided by measles and polio campaigns where possible. We only conducted this analysis for Lebanon because in the Lebanon survey respondents were asked more detailed questions about campaigns and the locations where children were vaccinated. In Jordan, the survey questions did not adequately differentiate recall of measles and polio vaccines provided through campaigns.

## 3. Results

A total of 1634 households in Jordan and 2165 in Lebanon were approached to participate in the survey. Of households approached in Jordan, 2.9% (*n* = 47) were not at home, 0.8% (*n* = 14) were already interviewed for this survey, and 1.4% (*n* = 23) declined to be interviewed. In Lebanon, 1.9% (*n* = 40) of approached households were not at home or not available, 0.2% (*n* = 4) were already interviewed for the survey, 0.05% (*n* = 1) were ineligible for participation, and 2.7% (*n* = 58) declined to be interviewed. The final samples included 1550 households in Jordan (with 9580 household members; response rate of 94.7%.) and 2062 households (1376 Syrian refugee and 686 host Lebanese households) in Lebanon (overall response rate of 93.6%). In both countries, survey respondents were predominantly female (61.7%, CI: 58.6–64.8 of refugees in Jordan; 59.3%, CI: 55.9–62.78 of refugees in Lebanon). The average age of refugee responses was also similar in both countries (Jordan mean = 38 years, median = 36, range: 15–95; Lebanon mean = 36, median = 34; range: 16–95). Within our sample, 88.1% (CI: 86.3–89.8) of the Syrian refugee households in Jordan, and 75.9% (CI: 73.2–78.5) of the Syrian refugee households in Lebanon were registered with UNHCR.

### 3.1. EPI Cards

Of all households visited, 24.3% of households in Jordan (*n* = 376) and 27.9% in Lebanon (*n* = 384) had a child aged 12–23 months. Among these households, 55.1% (*n* = 207) and 46.6% (*n* = 179) of respondents were able to present the child’s EPI card; 19.4% and 22.4% said the child had an EPI card but the card was not available; 23.4% and 26.3% said the child did not have an EPI card; and 2.1% and 4.7% of respondents did not know whether the child had an EPI card or not. No significant differences in card availability were observed by geographical region within either Jordan (*p* = 0.606) or Lebanon (*p* = 0.199).

### 3.2. Routine Vaccination Rates

Table 1 presents three figures for each vaccination. First, we calculated the proportion of children vaccinated using children 12–23 months with an EPI card whose card confirms they received the vaccine as the numerator and children 12–23 months with an EPI card as the denominator (abbreviated in our table as “card only/children with cards”). Second, we used children 12–23 months with an EPI card whose card confirms they received the vaccine as the numerator and all children 12–23 months as the denominator (abbreviated as “card only/all children”). Third, we used children 12–23 months with an EPI card whose card confirms they received the vaccine or who the respondent recalls as having received the vaccine as the numerator and all children 12–23 months as the denominator (abbreviated as “card and recall/all children”). Only a minority of children in both countries were fully immunized, having received BCG, measles, polio 1-3 and DPT 1-3 in Jordan, and measles, polio 1-3 and DPT 1-3 in Lebanon (BCG was not part of the Lebanon EPI schedule at the time of the survey). In Jordan, the fully immunized rate was 38.6% (CI: 31.6–46.2) when estimated using “card only/children with cards”; 21.3% (CI: 17.2–26.1) using “card only/all children”; and 24.5% (CI: 19.9–29.7) using “card and recall/all children”. In Lebanon, the fully immunized rate was 20.1% (CI: 15.0–26.5) using “card only/children with cards”; 9.4% (CI: 6.8–12.8) using “card only/all children”; and 12.5% (CI: 9.6–16.1) using “card and recall/all children”.

### 3.3. Difficulties Obtaining Vaccinations

Respondents were asked whether they experienced difficulties obtaining vaccinations for their children. In Jordan, 66.5% (CI: 60.9–71.6) of respondents said they experienced no difficulties; 9.6% (CI: 6.6–13.7) said they did not know where or when to take their child for vaccinations; 5.1% (CI: 3.3–7.7) said the vaccination location was too far away; and 4.3% (CI: 2.7–6.8) said they experienced long wait times at facilities. In Lebanon, 59.1% (CI: 53.7–64.3) said they experienced no difficulties; 10.2% (CI: 7.5–13.6) said they did not know where or when to take their child for vaccinations; 7.8% (CI: 5.5–11.0) said the vaccination location was too far away; and 4.9% (CI: 3.2–7.6) said vaccines were not available. There were no significant differences in vaccination difficulties by geographical region within Jordan (*p* = 0.803) or Lebanon (0 = 0.247).

### 3.4. Location of Vaccination (Lebanon Only)

For the survey in Lebanon, respondents were asked more detailed questions on vaccination campaigns and the location where the child was vaccinated. Among children who were vaccinated for measles through a campaign, 33.3% (CI: 25.7–42.0) were vaccinated at home and 66.7% (CI: 58.0–74.3) were vaccinated in a primary health care center. Most refugees vaccinated for polio through a campaign did so at a primary health care center (55.4%, CI: 48.3–62.3) or at home (36.5%, CI: 29.9–43.6) and a small proportion (8.1%, CI: 5.5–11.9) was vaccinated at the UNHCR registration office.

Supplementary analysis for Lebanon: Combined routine and campaign vaccination rates for measles and polio among children 12–23 months of age.

More than half of Syrian refugee children in Lebanon (59.4%, CI: 54.2–64.4) were reported to have received a measles vaccination through a campaign, and 68.2% (CI: 62.6–73.4) were protected from measles through a combination of routine and supplementary doses. (For these and the following estimates we used the “card and recall/all children” calculation approach, described above.) In our estimate, supplemental vaccination of measles boosted vaccination coverage by at least 25 percentage points over that provided by routine services alone (42.4%).

A similar proportion of Syrian refugee children (50.7%, CI: 39.2–62.1) were reported to have received polio vaccination through a campaign in Lebanon. We estimate the proportion of children protected against polio infection through 3 or more doses by calculating the percent of children who received either Polio 1 or Polio 2 through routine vaccination services and who received a campaign dose. We assume that some children reported to have received a campaign dose received both campaign doses (30 days apart) and some only received one of two. Although only 29.9% of children were reported to have received 3 doses of polio vaccine through routine vaccination services, we estimate that between 39.3% and 46.8% of Syrian refugee children aged 12–23 months of age are protected against polio infection in Lebanon. It appears that supplementary vaccination of polio boosted the percent of children protected against polio infection by at least 10–15 percentage points.

## 4. Discussion

In our surveys, the proportion of Syrian refugee children age 12–23 months who were documented or reported to be fully immunized through routine vaccination services was low: only 24.5% and 12.5% in Jordan and Lebanon, respectively. However, several mass vaccination campaigns were conducted in Jordan and Lebanon in 2013 and 2014 for measles and polio. During these campaigns, vaccinations were not recorded on children’s EPI cards, meaning that some children whose card did not show a measles or polio vaccination may have, in reality, received the vaccination. We estimate that repeated campaigns may boost the fully vaccinated coverage 10–15% or more. Even so, these rates are low. Such low coverage rates expose populations to heightened risk of outbreaks, putting children’s lives at risk and setting back global efforts to eradicate the diseases [5].

In Lebanon, 68.2% of Syrian refugee children were documented or reported to have received a measles vaccination, with only 42.4% of children receiving measles vaccine through routine services. These measles vaccination coverage rates are in between those of other surveys in Lebanon around the same time. One study in 2014 found that 78% of children aged 9–59 months had received at least one dose of measles vaccine [21]. Another study in 2015 documented a measles vaccination coverage rate of 59% among children 12–23 months of age, with coverage rates about 10% higher among children 24–48 months of age [22]. This suggests that vaccination coverage rates may vary significantly between surveys of children 12–23 months of age compared to surveys of children 9–59 months of age, and that age disaggregation is helpful.

In Jordan, 63.0% of Syrian refugee children in non-camp settings were documented or reported to have received measles vaccine in our survey. This rate is about 20% lower than other surveys in Jordan in the 2014–2015 time period. UNHCR found that 87% (in 2014) and 82% (in 2015) of Syrian refugee children in Jordan aged 9–59 months had received at received at least one dose of measles vaccine [23]. It may be that the age group surveyed here (12–23 months) had lower vaccination rates than the average among Syrian refugee children 9–59 months of age as documented above [22].

We found that only 33% of Syrian refugee children in Jordan, and between 39% and 47% of Syrian refugee children in Lebanon, ages 12–23 months, had evidence that they were protected against polio disease with three or more doses of polio vaccine. With multiple supplementary polio campaigns in Jordan and Lebanon, it is likely that rates of protection against polio disease was higher (perhaps 10–15 percentage points), but still too low to prevent outbreaks in the presence of polio virus. It is also likely that children in older age groups had higher levels of protection in that period, from either exposure to polio virus in earlier years, vaccine virus in the environment, and/or exposure to more campaigns. These findings suggest, however, that frequent campaigns are needed until more evidence of improved routine vaccination services is available.

While we believe our findings represent vaccination coverage rates that were indeed low, other surveys in the 2014–2015 time period reported higher vaccination rates. These other surveys used different sampling methodologies and sampled children of different ages, which may explain the some of the divergence, along with undocumented doses received during campaigns. We describe these issues in more detail below—not only to better explain our results, but to characterize inherent challenges with measuring vaccination rates among refugee and displaced populations.

### 4.1. Low Coverage of EPI Cards

In most household surveys, measuring vaccination coverage involves inspecting EPI cards or home-based records. However, in our studies, only 55.1% and 46.6% of children in Jordan and Lebanon had EPI cards. Because card coverage in our studies was so low, we faced a logical difficulty in calculating estimates. Three options were available to us (each reported in our results): (1) to use the number of children with cards as the denominator (likely introducing positive bias, because children with EPI cards are more likely to have received a vaccination, by virtue of the fact that they have a card); (2) to use all children as the denominator, but only include in the numerator those children whose vaccination was recorded on a card (likely introducing negative bias, because some children without cards may have, in fact, had the vaccination, for example during a campaign); and (3) to use all children as the denominator, and allow observations based on mother’s recall to be included in the numerator (likely introducing error due to recall bias). Typically, “card only/all children” is the conservative approach, as it guards against positive bias introduced by mother’s recall. But in this case, where families could have good reasons for not having an EPI card with them, it may not be the most representative. The “card and recall/all children” formulation may therefore be the better approach.

The issue of EPI card unavailability is likely not limited to the Syria conflict. Displaced families who have to leave their homes quickly, or have their homes destroyed, may not be able to bring vaccination cards with them. We therefore need consensus on how best to measure vaccine coverage in populations who are unlikely to maintain EPI cards. We know already that reliability of mothers’ recall may vary due to factors including information received or understood at the time of vaccination; the recall period; the complexity of the vaccination schedule; the interviewer’s demeanor, skills, use of language, and recording accuracy; and the length of the questionnaire and interview fatigue [24,25,26]. When the original EPI survey was introduced, vaccination schedules included fewer vaccines, and vaccines were given by different routes of administration (oral vs. injection) or at different locations on the body. There are now more vaccines and several new vaccines are given in the same parts of the body or via the same route of administration and may vary by country, adding complexity for questionnaire design and for respondents to recall vaccinations [10]. The updated EPI vaccination coverage survey methodology includes a module, where feasible, to double check vaccination coverage against local clinic records (fixed or outreach) for children without cards but reported vaccination [27]. This may be difficult or impractical in some settings, but could also serve to validate coverage estimates where EPI card availability is low. Measuring antibody titers could also provide more accurate estimates of immunity, though again this may be impractical, and since titers may be positive if a child had the disease, it would not be possible to differentiate between children who received a vaccine and who contracted the disease.

### 4.2. Campaign Coverage

A further complication is that vaccinations received in national campaigns are not always recorded on EPI cards. In Jordan and Lebanon, several measles and polio campaigns were conducted in the years preceding our surveys [28,29,30,31]. It is likely that some children in our survey whose EPI card did not show a measles or polio vaccination did in fact receive the vaccination through a campaign. As often occurs, these vaccination campaigns in Jordan and Lebanon were immediately followed by validation studies to estimate their reach and achieved coverage [28,29]. The fact that such validation studies are conducted so soon after the campaign allows for improved methodology, due to the shorter recall period or observation of a campaign indicator (e.g., gentian violet on a finger), but these studies do not estimate coverage of vaccines not administered in the campaign. There is still a need for cross-sectional surveys, or other methods, to estimate the proportion of children receiving all scheduled vaccinations at a given point in time, and/or immunized against key diseases.

### 4.3. Sampling Comparable Age Groups

Our survey enabled relatively large samples of children 12–23 months of age (376 in Jordan and 384 in Lebanon) by surveying 1550 refugee households in Jordan and 1376 refugee households in Lebanon. In the future, researchers may wish to broaden the age range, with sufficient sample sizes in the 12–23 month age group, to report both coverage among both children 12–23 months, and children 9–59 months of age. This recommendation is aligned with recent calls to expand the target age range for vaccination in emergency settings [9].

### 4.4. Limitations

Every attempt was made to create a robust study design and implement it with care, yet as in all studies, it is necessary to consider limitations.

With respect to sampling, reliance on UNHCR registration data may have resulted in sampling bias if the geographic distribution of registered and unregistered households differed. Reallocation of Lebanon clusters in areas controlled by militarily and political factions where permission to conduct the survey was not secured, specifically in the southern suburbs of Beirut and northern areas of Bekaa, resulted in a large area of the country being excluded; the remaining area included only 53% of registered Syrian refugees in the country and thus is not representative of the entire Syrian refugee population in Lebanon.

The second-stage referral process, choosing households within clusters, also presents the potential for bias in both surveys, as respondents may not have always referred to the nearest household and instead to friends, though referral procedures and small clusters size may have attenuated within-cluster similarities and the associated design effect. Replacement sampling, which was done for logistical purposes, also could contribute to bias if there are systematic differences between households where no one was at home compared with those interviewed.

The third-stage of sampling, randomly selecting eligible children in households with more than one child aged 12–23 months, also introduces potential error. We included a maximum of one child per household in our sample. The fact that some households had more than one child aged 12–23 months could introduce statistical error in our estimates, though we expect this error to be negligible. Moreover, by including only one child per household, we minimize the bias introduced by clustering of children within households.

Although our final sample included 376 children 12–23 months of age in Jordan and 384 in Lebanon, the study was neither designed nor powered a priori to estimate vaccination coverage in children 12–23 months of age.

Finally, interviews were conducted by Lebanese in Lebanon and Jordanians in Jordan which could have resulted in a higher refusal rate, hesitance, or influence on the part of Syrian refugees in responding to certain questions than if interviews had been conducted by Syrians.

## 5. Conclusions

The vaccination coverage estimates among Syrian refugee children in Jordan and Lebanon generated in these surveys are low but may underestimate the proportion of children protected from vaccine-preventable diseases that are included in supplementary vaccination campaigns but not documented on EPI cards. Nonetheless, ensuring sufficient vaccination coverage for refugee populations should be a high priority.

A sizeable proportion of households in Jordan (33.5%) and Lebanon (40.1%) reported difficulties obtaining vaccinations. If we hope to increase vaccine coverage, concerted efforts are required to address these difficulties, ensuring that the local immunization clinics are known and accessible to refugee families. We should also ensure that vaccinations are provided to refugees free of charge, as cost has been shown to be the primary barrier to care seeking for Syrian refugee children in both Jordan and Lebanon [32,33]. Vaccination efforts could establish outreach and mobile services targeting areas populated by Syrian refugees and be designed to increase education about vaccination services, particularly as the majority of refugees in both countries live in non-camp settings. Outreach services could prioritize locations where pockets of unvaccinated children are estimated to be more likely [34].

Ensuring sufficient vaccination coverage also requires accurate data, yet the results of this study raise methodological issues for estimating vaccination coverage among refugees and displaced populations. Current methods to measure coverage—particularly of non-campaign, routinely administered vaccines—are problematic in humanitarian settings. New methods are needed. To ensure accurate understanding of vaccination coverage in displaced populations, we need research on: validity of recall methods, greater links between campaigns and routine immunization programs, commitment to accurate research methods, and sampling of hard-to-reach populations.

## Figures and Tables

**Table 1:** Vaccination Rates Among Syrian Refugees ages 12–23 months in Jordan and Syria.

Vaccine		Jordan			Lebanon		
		%	95% CI	*n/N*	%	95% CI	*n/N*
BCG							
Card only/children with cards		93.2	[89.0, 95.9]	193/207	22.3	[16.3, 29.9]	40/179
Card only/all children		51.3	[46.4, 56.3]	193/376	10.4	[7.4, 14.4]	40/384
Card and recall/all children		83.2	[79.4, 86.5]	313/376	29.2	[25.0, 33.7]	112/384
Measles							
Card only/children with cards		66.7	[60.1, 72.6]	138/207	50.8	[43.9, 57.8]	91/179
Card only/all children		36.7	[31.8, 41.9]	138/376	23.7	[19.6, 28.4]	91/384
Card and recall/all children		63.0	[57.8, 68.0]	237/376	42.4	[37.0, 48.1]	163/384
Polio							
Polio 0	Card only/children with cards	33.8	[26.9, 41.5]	70/207	41.9	[34.8, 49.4]	75/179
	Card only/all children	18.6	[14.8, 23.2]	70/376	19.5	[15.8, 23.9]	75/384
	Card and recall/all children	31.4	[26.5, 36.7]	118/376	40.4	[35.3, 45.7]	155/384
Polio 1	Card only/children with cards	80.2	[73.8, 85.3]	166/207	87.7	[81.7, 91.9]	157/179
	Card only/all children	44.1	[39.1, 49.4]	166/376	40.9	[35.5, 46.5]	157/384
	Card and recall/all children	52.1	[46.9, 57.3]	196/376	55.7	[50.2, 61.2]	214/384
Polio 2	Card only/children with cards	71.5	[64.6, 77.5]	148/207	69.3	[61.3, 76.2]	124/179
	Card only/all children	39.4	[34.3, 44.6]	148/376	32.3	[27.1, 37.9]	124/384
	Card and recall/all children	46.5	[41.2, 52.0]	175/376	45.8	[40.0, 51.8]	176/384
Polio 3	Card only/children with cards	51.2	[44.2, 58.2]	106/207	46.9	[39.3, 54.8]	84/179
	Card only/all children	28.2	[23.8, 33.1]	106/376	21.9	[17.6, 26.9]	84/384
	Card and recall/all children	32.7	[27.9, 37.9]	123/376	29.9	[25.3, 35.0]	115/384
DPT							
DPT 1	Card only/children with cards	89.4	[83.2, 93.5]	185/207	66.5	[59.0, 73.2]	119/179
	Card only/all children	49.2	[43.9, 54.5]	185/376	31	[25.9, 36.6]	119/384
	Card and recall/all children	67.6	[62.3, 72.4]	254/376	46.9	[41.2, 52.6]	180/384
DPT 2	Card only/children with cards	82.1	[74.8, 87.7]	170/207	56.4	[49.2, 63.4]	101/179
	Card only/all children	45.2	[39.9, 50.6]	170/376	26.3	[21.6, 31.6]	101/384
	Card and recall/all children	60.6	[55.0, 66.0]	228/376	35.7	[30.6, 41.1]	137/384
DPT 3	Card only/children with cards	71	[63.8, 77.3]	147/207	36.3	[30.0, 43.2]	65/179
	Card only/all children	39.1	[33.9, 44.5]	147/376	16.9	[13.4, 21.2]	65/384
	Card and recall/all children	49.2	[43.3, 55.1]	185/376	21.4	[17.4, 25.9]	82/384
Fully Immunized		BCG, measles, polio 1–3, DPT 1–3			Measles, polio 1–3, DPT 1–3		
Card only/children with cards		38.6	[31.6, 46.2]	80/207	20.1	[15.0, 26.5]	36/179
Card only/all children		21.3	[17.2, 26.1]	80/376	9.4	[6.8, 12.8]	36/384
Card and recall/all children		24.5	[19.9, 29.7]	92/376	12.5	[9.6, 16.1]	48/384
